# Factors associated with unquantifiable total HIV-1 DNA in peripheral blood in persons living with HIV: An observational study

**DOI:** 10.1016/j.jve.2024.100370

**Published:** 2024-03-28

**Authors:** Aurélie Ram, Vanessa Rascon Velasco, Gilbert Mchantaf, Véronique Avettand-Fénoël, Jean-Paul Viard

**Affiliations:** a Service d’Immuno-infectiologie, Hôpital Hôtel-Dieu, Assistance Publique-Hôpitaux de Paris, Paris, France; b Laboratoire de Virologie, Hôpital Cochin, Assistance Publique-Hôpitaux de Paris, Paris, France; c Université Paris Cité, School of Medicine, France; d Inserm U1016, CNRS UMR8104, Equipe Retrovirus, Infection, Latence, Institut Cochin, Paris, France

**Keywords:** HIV-1 infection, Viral reservoir, HIV -1 DNA

## Abstract

**Background:**

The human immunodeficiency virus type 1 (HIV-1) cannot be eradicated even with suppressive antiretroviral therapy because its retrotranscribed genome integrates into the DNA of host cells, creating a long-term reservoir. Quantification of total HIV-1 DNA in peripheral blood is a biomarker of this reservoir that can predict progression of the infection, treatment response, and HIV-1-related complications. A deeper understanding of the reservoir may help develop a cures.

**Objective:**

This study aimed to characterize persons living with HIV-1 (PLWH) with unquantifiable total HIV-1 DNA in blood (below the quantification threshold) and identify associated factors.

**Methods:**

We have conducted a retrospective observational study. During the study period, all PLWH who had total leukocyte-associated HIV-1 DNA measured by quantitative PCR were included. We have isolated a population of participants with HIV-1 DNA levels below the quantification threshold (40 copies/10^6^ leukocytes).

**Results:**

Out of 1094 patients analysed, 62 had unquantifiable and 1032 quantifiable HIV-1 DNA levels in blood. We have found that those with unquantifiable HIV-1 DNA had a higher CD4 T cell nadir (p = 0.006) and a lower viral load zenith (p < 0.001). Multivariate analyses showed that initiation of treatment in primary infection was the only protective factor against HIV-1 DNA quantifiability, the odds of HIV-1 DNA quantifiability decreased by 82% in those treated within 30 days of infection, after controlling for other factors.

**Conclusion:**

Our research highlights the importance of an early start of anti-retroviral therapy to limit the size of the HIV-1 reservoir, as receiving treatment during primary infection was found as the only protective factor against quantifiability of HIV-1 DNA in blood.

## 1 Introduction

In persons living with HIV-1 (PLWH), combined anti-retroviral therapy (cART) can lead to undetectable HIV-1 RNA viral load in blood. However, HIV-1 cannot be eradicated because, after reverse transcription, it integrates its genetic material into the DNA of host cells and forms a latent and persistent reservoir. This mechanism explains the rebound of viral replication in plasma when cART is discontinued. The reservoir persists for decades and is a barrier to curing HIV-1 infection.^1^ The DNA reservoir decreases slowly, even with long-term suppressive treatment, with an estimated median half-life of 44 months. On cART, it is estimated that it would take between 50 and 70 years to eradicate the entire reservoir, which appears unlikely to be achieved.^2^ Memory CD4^+^ T cells are the main HIV-1 reservoir.^3^ HIV-1 DNA exists in a variety of molecular configurations. The integrated form, known as the provirus, represents the main long-term reservoir and is implicated in viral relapse after ART interruption and is capable of producing virions infecting new cells. However, HIV-1 DNA can undergo mutations that makes it defective and unable to generate replication-competent viruses. Still, defective proviruses can generate viral transcripts and proteins that maintain immune activation in the host and contribute to HIV-1 pathogenesis. The non-integrated HIV-1 DNA exists in linear and circular forms as episomal 1- and 2-long terminal repeat (LTR). The non-integrated forms can contribute to transcription, but cannot produce virions on their own. Evidence suggests that the total HIV-1 DNA mainly reflects the level of integrated forms in individuals receiving cART.^4^^,^^5^ Several methods have been used to estimate the reservoir, including quantitative coculture assays, which are costly, time-consuming, technique-dependent, and require a substantial volume of blood. Total HIV-1 DNA quantification in peripheral blood mononuclear cells (PBMCs) by real-time PCR is a reliable, standardized, reproducible, and sensitive technique that has been widely used in large cohorts of patients and explored as an estimate of the reservoir.^5^

## 2 Methods

This retrospective, observational study was conducted in PLWH cared for at the Hôtel-Dieu Hospital in France. We have included all PLWH who had a quantification of total HIV-1 DNA level from January 2017 to June 2021. When a patient had had several HIV-1 DNA quantifications, the latest measure was considered. Total HIV-1 DNA was quantified in blood samples with the Generic HIV DNA Cell kit, according to the manufacturer's instructions (Biocentric, Bandol, France).^6^ Results were expressed as the number of HIV-1 DNA copies/10^6^ leukocytes. The threshold of detection of the technique was 40 HIV-1 DNA copies/10^6^ leukocytes. The outcome was interpreted in analyses as a dichotomous variable: PLWH with unquantifiable HIV-1 DNA, and PLWH with quantifiable HIV-1 DNA in blood, with no need to calculate the number of copies/10^6^ PBMCs.

Medical records were reviewed to collect demographic and clinical data using a standardized, specific form. Demographic data extracted included sex assigned at birth, mode of HIV-1 infection (sexual, blood, or mother-to-child transmission), and region of birth. Clinical data extracted included HIV-1 related characteristics such as subtype, CDC stage, CD4 T cell nadir, HIV-1 RNA zenith, latest CD4 T cell count, latest CD4/CD8 T cell ratio, known duration of infection, and whether or not the patient started cART within 30 days after primary infection.

The CD4 T cell count and plasma HIV-1 RNA load were measured according to standard procedures. HIV-1 RNA was assessed with the Abbott m2000 assay, with a limit of quantification of 20 copies/ml.

### 2.1 Statistical analyses

Characteristics of patients were expressed as median (IQR) for continuous variables and frequency (%) for categorical variables. Wilcoxon's rank sum test and Fisher's exact test were used for the comparison of values between groups with quantifiable and unquantifiable HIV-1 DNA for continuous and categorical variables, respectively. A logistic regression model was performed to estimate the unadjusted and adjusted odds ratios (OR) along with their respective 95% confidence intervals. The significance level was set at 0.05 for all tests, and all analyses were completed with Windows R software version 4.2.0.

## 3 Results

### 3.1 Patient characteristics

Table 1 summarizes the baseline characteristics of the 1094 PLWH included in the study, among whom 62 had unquantifiable HIV-1 DNA (<40 HIV-1 DNA copies/10^6^ leukocytes) and 1032 patients had quantifiable HIV DNA. The median age of the overall population was 55 years (46.0–61.0). Seventy-six percent of our population were men, and 60% were born in Western and Central Europe or North America. The patients with unquantifiable HIV-1 DNA had a higher CD4 T cell nadir (p = 0.006), lower viral load zenith (p < 0.001), and had more frequently started treatment during primary infection (13% vs. 2.3 %, p < 0.001) compared to those with quantifiable HIV-1 DNA.

**Table 1:** Patient characteristics according to HIV-1 DNA quantifiability groups.

Characteristic	Overall	Unquantifiable HIV-1 DNA	Quantifiable HIV-1 DNA	Missing	p-value^b^
	N = 1,094^a^	N = 62^a^	N = 1,032^a^		
**Age** (years)	55.0 (46.0–61.0)	53.5 (37.8–61.0)	55.0 (47.0–61.0)	0 (0%)	0.245
**Sex assigned at birth**				0 (0%)	0.879
Male	832 (76%)	48 (77%)	784 (76%)		
Female	262 (24%)	14 (23%)	248 (24%)		
**Region of birth**				1 (<0.1%)	>0.999
Western and Central Europe and North America	653 (60%)	37 (60%)	616 (60%)		
Sub-Saharan Africa	283 (26%)	16 (26%)	267 (26%)		
North Africa and Middle East	52 (4.8%)	3 (4.8%)	49 (4.8%)		
Other	105 (9.6%)	6 (9.7%)	99 (9.6%)		
**Route of HIV acquisition**				60 (5.5%)	0.481
Sexual transmission: Men who have sex with men (MSM)	556 (54%)	33 (56%)	523 (54%)		
Sexual transmission: Excluding MSM	360 (35%)	19 (32%)	341 (35%)		
Blood transmission	80 (7.7%)	3 (5.1%)	77 (7.9%)		
Mother-to-child transmission	38 (3.7%)	4 (6.8%)	34 (3.5%)		
HLA-B*57:01				484 (44%)	0.413
Present	577 (95%)	30 (91%)	547 (95%)		
Absent	33 (5.4%)	3 (9.1%)	30 (5.2%)		
**HIV-1 group/subtype**				225 (21%)	0.002
M/B	567 (65%)	23 (64%)	544 (65%)		
M/non B	300 (35%)	11 (31%)	289 (35%)		
O	2 (0.2%)	2 (5.6%)	0 (0%)		
**CDC stage at diagnosis**				0 (0%)	0.203
A	766 (70%)	50 (81%)	716 (69%)		
C	211 (19%)	8 (13%)	203 (20%)		
B	117 (11%)	4 (6.5%)	113 (11%)		
**Latest HIV-1 viral load**				0 (0%)	0.501
<20 copies/mL	891 (81%)	53 (85%)	838 (81%)		
≥20 copies/mL	203 (19%)	9 (15%)	194 (19%)		
**Zenith HIV-1 viral load** (copies/mL)	55,991.5 (7,691.5–190,000.0)	8,989.0 (655.8–50,362.3)	62,150.0 (9,909.3–203,500.0)	0 (0%)	<0.001
**CD4 T cell nadir** (cells/μL)	250.0 (126.3–392.8)	290.5 (174.3–449.8)	248.0 (124.0–387.3)	0 (0%)	0.060
**Latest CD4 T cells** (cells/μL)	608.5 (442.0–795.8)	568.0 (446.0–738.3)	614.0 (441.8–797.0)	0 (0%)	0.398
**Latest CD4/CD8 T cell ratio**	0.9 (0.7–1.3)	0.9 (0.7–1.3)	0.9 (0.7–1.3)	9 (0.8%)	0.803
**Known duration of HIV-1 infection** (years)	17.9 (9.0–25.9)	12.7 (4.8–24.0)	18.1 (9.4–25.9)	0 (0%)	0.048
**Initiation of cART during primary infection**					
No	1,062 (97%)	54 (87%)	1,008 (98%)	0 (0%)	<0.001
Yes	32 (2.9%)	8 (13%)	24 (2.3%)		

a Median (IQR); n (%).

b Wilcoxon rank sum test; Fisher's exact test.

### 3.2 Predictors of HIV-1 DNA quantifiability

Multivariate analyses showed that prompt initiation of treatment in primary infection was a protective factor against having quantifiable HIV-1 DNA level when controlling for the other factors. Additionally, the odds of having quantifiable HIV-1 DNA dropped by about 82% in patients who had been treated within 30 days after infection. CDC stage at initiation of treatment, viral load zenith, CD4 T cell nadir, or duration of HIV-1 infection did not show a significant association with HIV-1 DNA detectability in the model (Table 2).

**Table 2:** Logistic regression analyses of the odds of HIV-1 DNA quantifiability in PLWH (N = 1,094).

	Univariate analysis		Multivariate analysis	
Characteristics	uOR (95% CI)^a^	p-value	aOR (95% CI)^a^	p-value
**Age** (years)	1.02 (1.00–1.04)	0.127	1.00 (0.98–1.03)	0.788
**Sex assigned at birth (F)**	1.08 (0.60–2.07)	0.795	0.93 (0.51–1.81)	0.830
**CDC stage at diagnosis**				
A	0.56 (0.24–1.14)	0.141	0.74 (0.31–1.60)	0.472
B	1.11 (0.34–4.25)	0.863	1.17 (0.35–4.50)	0.809
**Zenith HIV-1 viral load** (copies/mL)	1.00 (1.00–1.00)	0.711	1.00 (1.00–1.00)	0.498
**CD4 T cell nadir** (/μL)	1.00 (1.00–1.00)	0.070	1.00 (1.00–1.00)	0.686
**Known duration of HIV-1 infection** (years)	1.03 (1.00–1.05)	**0.032**	1.01 (0.98–1.04)	0.479
**Initiation of ART during primary infection**	0.16 (0.07–0.40)	**<0.001**	0.19 (0.08–0.49)	**<0.001**

a OR = Odds Ratio (u: unadjusted, a: adjusted), CI = confidence interval.

## 4 Discussion

Although numerous studies have been conducted on the dynamics, associated markers, and clinical impact of HIV-1 DNA, few have studied a population of PLWH with unquantifiable HIV-1 DNA to confirm the observed trends. To our knowledge, only the study done by Nozza et al.^7^ focused on a specific population of PLWH with undetectable HIV DNA in the blood, yet only PLWH treated in the chronic phase of the infection were examined in this study. This study found that unquantifiable HIV DNA was associated with a lower HIV RNA zenith, a shorter time between HIV diagnosis and initiation of cART, and a greater CD4 nadir.^7^ Our research confirms that PLWH with unquantifiable HIV DNA had a lower viral load zenith and a higher CD4 nadir. Moreover, only few differences were observed between the groups of unquantifiable and quantifiable HIV DNA, as receiving treatment during primary infection was seen as the only protective factor against HIV DNA quantifiability. This finding is comparable to Ngo-Giang-Huong et al.'s study where they also demonstrated that the decrease in HIV DNA after one year of treatment was greater when treatment was initiated during the primary infection phase rather than in the chronic infection phase.^8^ Another study also showed that the earlier cART was initiated, the faster HIV DNA levels decreased, resulting in a lower median reservoir level after five years of cART in PLWH who were treated early.^9^ Lastly, Gianela et al.,^10^ also suggested that this benefit persists even after discontinuation of antiretroviral therapy, and that initiating treatment within two months of an HIV primary infection diagnosis reduced plasma HIV DNA levels for up to a year after treatment suspension. These findings highlight the importance of treating during the primary infection phase to minimize the impact on reservoir seeding. The benefits of early treatment could be linked to reducing the settlement of stable integrated forms, the proportion of which among total HIV DNA increases during the first year of infection.^11^^,^^12^ Conversely, several studies question the significance of quantifying total HIV DNA and whether the DNA detected is representative of intact proviral DNA, competent for viral replication. To quantify the competent reservoir as precisely as possible and to exclude defective proviruses, different techniques have been developed, including IPDA (Intact Proviral DNA Assay) and Q4PCR (Quadruplex PCR). When these methods are used together, they may provide better insight into the size of the HIV reservoir as shown by the study conducted by Kinloch et al., where levels of IPDA correlate with the quantification of total and integrated DNA in the plasma of patients receiving cART.^13^ Another limitation of the reservoir's quantification is linked to the fact that HIV reservoirs are mainly found in tissues.^14^ Research has indicated that intact HIV proviral DNA persists in the brain at levels related to those detected in blood and lymphoid tissues^15^ and that a correlation between HIV DNA found in blood and rectal cells or lymph nodes exists.^16^ Despite these limitations, total HIV DNA level in blood has proven its significance in numerous clinical situations and should be considered as a clinically relevant marker.^5^ Other limitations of our study include its observational, retrospective design, with missing data, and the constraint to use routinely available variables, which precludes the possibility of addressing pathophysiology issues (e.g., genetic factors).

In conclusion, our research highlights the importance of an early start of anti-retroviral therapy to limit the size of the HIV reservoir, as receiving treatment during primary infection was seen as the only protective factor against leukocyte-associated HIV DNA quantifiability.

## Funding

The authors have not declared a specific grant for this research from any funding agency in the public, commercial, or not-for-profit sectors.

## CRediT authorship contribution statement

**Aurélie Ram:** Writing – review & editing, Writing – original draft, Visualization, Validation, Methodology, Investigation, Data curation, Conceptualization. **Vanessa Rascon Velasco:** Writing – review & editing, Formal analysis, Data curation. **Gilbert Mchantaf:** Writing – review & editing, Data curation. **Véronique Avettand-Fénoël:** Writing – review & editing, Validation, Supervision, Data curation. **Jean-Paul Viard:** Writing – review & editing, Validation, Supervision, Methodology, Investigation, Data curation, Conceptualization.

## Declaration of competing interest

The authors declare that they have no known competing financial interests or personal relationships that could have appeared to influence the work reported in this article.

## Data Availability

Data will be made available on request.
